# The interplay of factors influencing the carbon footprint of hospital care—A causal mapping analysis of scientific reports

**DOI:** 10.1016/j.joclim.2025.100427

**Published:** 2025-03-12

**Authors:** L.H.J.A. Kouwenberg, D.S. Kringos, W.J.K. Hehenkamp, E.S. Cohen, N.H. Sperna Weiland

**Affiliations:** a Amsterdam UMC Location University of Amsterdam, Public and Occupational Health, Meibergdreef 9, Amsterdam, the Netherlands; b Centre for Sustainable Healthcare, Amsterdam UMC, University of Amsterdam, Amsterdam, the Netherlands; c Amsterdam Public Health, Quality of Care, Amsterdam, the Netherlands; d Amsterdam UMC Location Vrije Universiteit Amsterdam, Department of Obstetrics and Gynecology, De Boelelaan 1117, Amsterdam, the Netherlands; e Amsterdam UMC Location University of Amsterdam, Department of Anesthesiology, Meibergdreef 9, Amsterdam, the Netherlands

**Keywords:** Carbon footprint, Determinants, Causal mapping, Climate change, Hospital, Healthcare

## Abstract

**Introduction:**

Climate change threatens human well-being and planetary health, necessitating sector-wide transitions. Recent research has highlighted the carbon footprint of hospital care by identifying hotspots and mitigation areas, but key factors influencing these outcomes remain underexplored.

**Methods:**

This study used causal mapping of textual data to systematically evaluate scientific reports on the carbon footprint of hospital services and care pathways. The sample was drawn from a State-of-the-science literature review, focusing on quantitative reports on hospital services’ carbon footprint. Text fragments discussing factors influencing the carbon footprint were recorded, and variables and relationships were identified and visually mapped through iterative open, axial, and selective coding.

**Results:**

Twelve main factors influence four major domains of the carbon footprint of hospital services and care pathways. These factors are related to the volume of travel, facilities and equipment, consumables, waste disposal, and pharmaceuticals, and their carbon intensity. Over eighty subfactors were identified, including ten cross-cutting factors that affect multiple domains of the hospital care footprint.

**Conclusions:**

The carbon footprint of hospital care is a multifaceted and complex issue driven by multiple factors. Insight into these factors can inform targeted actions to reduce emissions. This study also improves the understanding of the causes of variability in carbon footprint outcomes of hospital care, which is important for the interpretation and transferability of results and conclusions in this rapidly growing field of research.

## Graphical abstract

**Figure:**
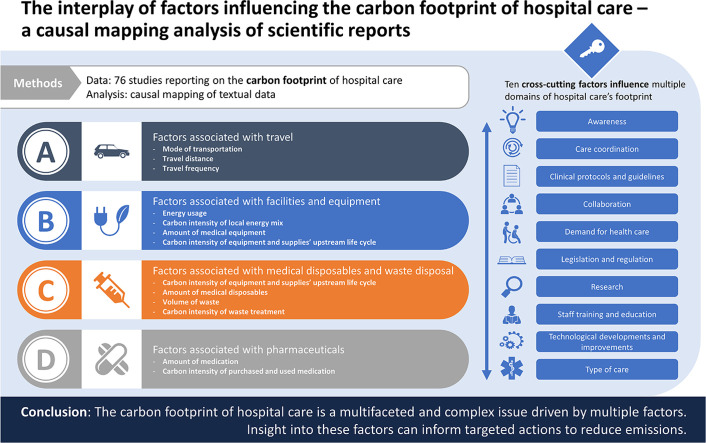

## 1 Introduction

Climate change poses a significant threat to human well-being and planetary health, requiring transitions across all sectors [1]. This includes the health care sector which has been identified as a major contributor to greenhouse gas emissions [[2], [3], [4]]. Several studies have examined the climate impact of health care at different levels: globally [4], at the level of national health care systems [3,5,6], at the hospital or department level [7,8], and at the level of care pathways or individual health care services [9]. These studies provide valuable insights into the carbon footprint of different alternatives and identify carbon hotspots, which can help target and prioritize mitigation strategies for a range of stakeholders, including policymakers, health care professionals, hospital administrators, suppliers, and patients.

Studies have demonstrated large variance in health care carbon footprints between countries, which can largely be attributed to country-specific characteristics, such as the carbon intensity of the energy system and health care expenditures [2,4]. Additionally, large variations in carbon footprint are observed at other levels of analysis, including care pathways and health care services. A state-of-the-science review showed that the carbon footprint of hospital services and care pathways varied considerably between services, medical specialties and settings [9]. For instance, the carbon footprint of cataract surgery differs across countries [10,11], and across different types of hospitals [12]. This variability can hinder the effective use of carbon footprint data, when designing and implementing mitigation strategies across different settings.

Despite progress in understanding the climate impact and carbon hotspots of specific hospital services in different contexts, a comprehensive understanding of the key factors and their relationships influencing the carbon footprint of hospital services and care pathways is still lacking. Some studies suggested a few driving factors, such as geographic location or the age of buildings [13], but a broad range of factors and mechanisms contributing to variations in carbon footprint results across different services, specialties and settings remain underexplored [14]. With the rapid growth of environmental impact studies in healthcare, there is an opportunity to learn from the direct and indirect factors influencing the carbon footprint identified in these scientific reports.

To design effective interventions aimed at reducing the climate impact of hospital care, it is essential to identify potential levers for change that stakeholders can act upon. Furthermore, exploring the factors influencing the carbon footprint of hospital care will enhance the understanding of this observed variation, which will also contribute to transferability of results and conclusions from one setting to another. Therefore, this study aims to identify and map the factors influencing the carbon footprint of hospital care, and their interrelationships, by addressing the research question: What type of factors influence the carbon footprint of individual hospital services and care pathways and how do they interact?

## 2 Methods

To identify influential factors and their interrelationships, literature on the carbon footprint of hospital services and care pathways was systematically evaluated. Causal mapping of textual data was employed, a recognized method for system modeling [15,16]. Causal mapping involves inductively coding qualitative ‘purposive’ text data to generate causal maps. Purposive text data arises from experts’ discussions on the system and problem under study, revealing the stakeholders’ mental models. In this study, qualitative data were derived from research articles that have studied the carbon footprint of hospital services or care pathways.

### 2.1 Study selection

The current study utilized the same articles previously identified in a state-of-the science literature review, conducted in January 2024 [9]. This review systematically identified studies on the carbon footprint of hospital service and care pathways from six different databases, additional grey sources, and the Healthcare LCA database [17]. The included studies reported quantitatively on the carbon footprint and hotspots of hospital services or care pathways. In contrast, excluded studies focused on entire hospitals or health care systems, were published before the year 2000, or consisted of reviews, opinion-based reports, commentaries, or editorials. Detailed selection criteria are provided in the original article [9].

### 2.2 Data extraction

From the included studies, the results and discussion sections were screened for text fragments discussing the (potential) influence of specific factors on the reported carbon footprint of hospital services or care pathways, defined as potential drivers or determinants of carbon footprint outcomes, such as energy sources, or waste management practices. Fragments were recorded if they suggested any factors potentially positively or negatively influencing the carbon footprint.

### 2.3 Data analysis

To analyze and build a causal map from qualitative text, a four-step approach was followed: 1) Identifying concepts and discovering themes in the data; 2) Categorizing and aggregating themes into variables; 3) Identifying relationships between aggregated variables; and 4) Transforming the coding dictionary into a visual map [15,16]. A grounded theory approach, involving iterative coding to generate theory from data, was employed [18]. Different coding methods were used in phases: open coding to break down, examine, compare, and categorize data; axial coding to reassemble and generalize open codes into overarching variables; and selective coding to integrate and synthesize categories into core categories and sub-systems [16]. For example, a text fragment stating that “large differences in scanner energy consumption have been reported, due to both differences in actual scanners as well as usage patterns. Because of this variability, absolute levels of emissions at other sites may differ” [19] was open coded as ‘Usage patterns influencing scanner energy consumption during use and in standby mode’, axial coded as ‘Utilization rate’, and selective coded as ‘Energy usage’.

In the fourth step, axial and selective codes and their interrelationships were visually mapped using Kumu software [20]. The direction of causal relationships was not indicated, as these were often unclear from the data. To minimize coder bias, interpretations of causal arguments not explicitly stated in the text were kept to a minimum. Finally, the map was further refined, by iteratively reassembling data elements and relationships with axial coding and selective coding, to integrate and synthesize the categories from previous steps into core categories and identify sub-systems.

During the analysis memos were written to summarize insights that arose during coding. One researcher (LHJAK) read and examined the results and discussion sections from primary studies and extracted relevant text fragments. The resulting codes and codebook were discussed with two other researchers (NHSW, DSK), to refine the codes and categories. Coding was done using Microsoft® Excel® (Version 2402 Build 17328.20550), and emerging themes were discussed among the authors. The coding scheme is presented in the Supplementary Materials.

## 3 Results

Analysis of the 76 included studies revealed twelve distinct main factors, grouped into four categories, which influence the carbon footprint and carbon emission hotspots of hospital services and care pathways (Table 1).

**Table 1:** Factors influencing the carbon footprint and carbon emission hotspots of hospital services and care pathways.

Category	Main factor
Travel	Mode of transportation
	Travel distance
	Travel frequency
Facilities and equipment	Energy usage
	Carbon intensity of local energy mix
	Amount of medical equipment
	Carbon intensity of equipment and supplies’ upstream life cycle
Medical disposables and waste disposal	Carbon intensity of equipment and supplies’ upstream life cycle
	Amount of medical disposables
	Volume of waste
	Carbon intensity of waste treatment
Pharmaceuticals	Amount of medication
	Carbon intensity of purchased and used medication

The main factors, related subfactors and their interrelationships are described and mapped in detail in the following section.

### 3.1 Factors related to the carbon footprint of travel

**Fig. 1:**
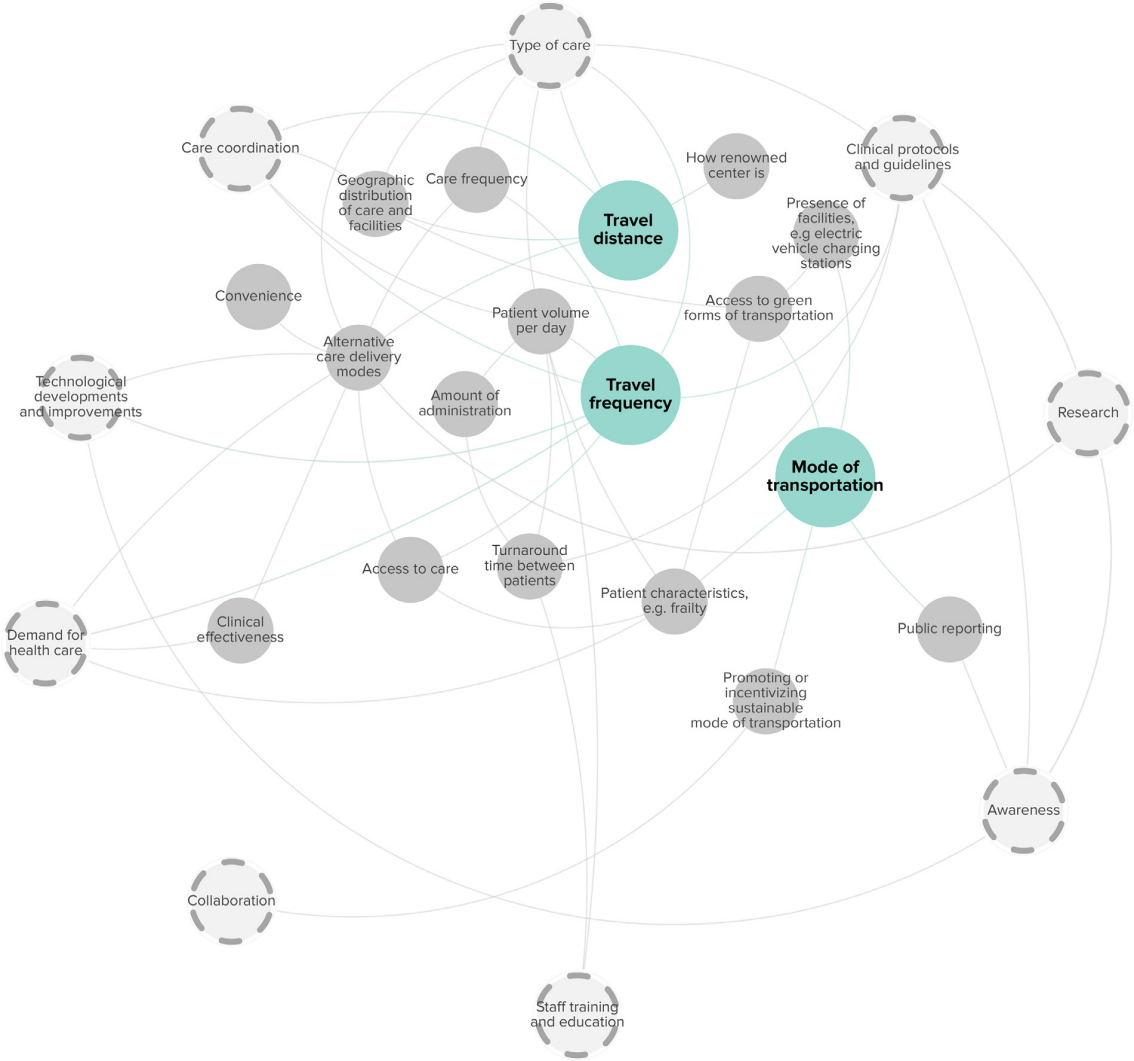
Factors associated with the carbon footprint of travel. Main factors are highlighted in blue. Dashed circles highlight the cross-cutting factors.

The carbon footprint due to travel of patients and hospital staff was influenced by three main factors: 1) Mode of transportation; 2) Travel distance; and 3) Travel frequency. Fig. 1 displays their related subfactors and interrelationships.

#### 3.1.1 Mode of transportation

The carbon emissions from transportation vary based on the mode used by patients, staff, and visitors. Green options such as buses or trains generally have lower emissions, but their use depends on access [[21], [22], [23], [24]], the geographic distribution of hospital facilities [8,21,25,26], and the presence of facilities such as bicycle-sharing programs and clean-energy-sourced electric vehicle charging stations [22]. Raising awareness about the carbon footprint of different transportation options through public reporting [27], and incentives can also encourage greener forms of transportation [12,23,28,29]. Finally, patients’ characteristics like frailty and disabilities also influence patients’ choices for different modes of transportation [23].

#### 3.1.2 Travel distance

The geographic distribution of care and facilities influences the distance travelled to receive hospital care [8,[24], [25], [26],[30], [31], [32], [33], [34], [35], [36], [37]], though coordination of care can mitigate this [24,31,35,38,39]. Alternative care delivery modes, such as telemedicine and treatments provided at-home can reduce travel needs [26,27,37,40,41], but they rely on the type of care [37], research [27], technical developments [41], and user convenience [30]. Finally, specialized types of care often require patients to travel longer distances compared to primary care [26,30,40].

#### 3.1.3 Travel frequency

Travel frequency is influenced by clinical protocols [25], the type, frequency and duration of care [8,25,26,39,42], and clinical effectiveness [30]. Care coordination can consolidate appointments on the same day, reducing patient travel [11,12,[43], [44], [45]]. Increasing daily patient volume could also decrease staff travel per case; however, feasibility depends on factors like the type of care, patient characteristics, turnaround time between patients, administrative demands, and staff training and education [12]. Table 2 provides a detailed description of the factors related to the carbon footprint of travel.

**Table 2:** Factors and subfactors linked with travel.

Category	Contributing factor	Subfactor	Reference(s)	Associated subfactor(s)	Reference(s)	Description
Travel	Mode of transportation	Access to green forms of transportation	[[21], [22], [23], [24]]	Geographic distribution of care and facilities & presence of facilities, e.g., electric vehicle charging stations	[8,21,22,25,26]	Access to green forms of transportation could facilitate people to travel by bus or train, but this accessibility also depended on the geographic distribution of facilities and hospitals’ proximity to these networks. The presence of bicycle-sharing programs and clean-energy-sourced electric vehicle charging stations could increase access to green forms of transportation.
				Patient characteristics, e.g., frailty	[23]	Different subfactors may be relevant for patients and staff, where patients’ access to green forms of transportation is also influenced by patient characteristics, such as the presence of frailty and disabilities.
		Awareness	[27]	Public reporting and research	[27]	The use of greener forms of transportation may be influenced by people's awareness about the carbon footprint of different modalities, which could be increased by public reporting of hospitals by displaying information and research findings in waiting rooms and on websites.
		Promoting or incentivizing sustainable mode of transportation	[12,23,28,29]	Collaboration	[12]	Active promotion and encouragement of greener forms of transportation may increase their use, for example with financial incentives for public transportation, created through collaboration between hospitals with local government.
	Travel distance	Alternative care delivery modes	[26,27,30,37,40,41]	Type of care, technological developments and improvements, research & convenience	[27,30,37,41]	Alternative care delivery modes, such as treatments provided at-home have the potential to improve access to care, and reduce travel distance, though not all types of care are suitable for other care delivery modes such as telehealth. Research findings could lead to the development of alternative care delivery modes. Their development also depends on technical developments and improvements, for example the development of new and portable hemodialysis devices and telemedicine solutions allowing patients to be treated from home, and convenience and ease of use also influenced their use.
		Geographic distribution of care and facilities	[8,[24], [25], [26],[30], [31], [32], [33], [34], [35], [36], [37]]	Care coordination	[24,31,35,38,39]	Travel distance was influenced by the geographic distribution of care and facilities. In rural areas, patients and staff were required to travel further to reach the nearest hospital, but the presence of local care facilities and coordination of care in these places could reduce this need.
		How renowned center is	[21]			Patients may travel further to renowned centers for certain treatments.
		Type of care	[26,30,40]			The distance that was travelled by patients was also related to the type of care provided, e.g., specialized care generally requiring patients to travel longer distances, and primary care provided close to patients’ homes.
	Travel frequency	Access to care	[25]			Reduced access to care during the pandemic also led to fewer visits, because of fear of disease transmission.
		Care coordination	[11,12,[43], [44], [45]]			Frequency of travel was related to care coordination, for example the planning of different appointments and treatments on the same day.
		Demand for healthcare	[30]	Clinical effectiveness	[30]	The effectiveness of care influenced demand for additional visits.
		Duration of care	[25,26,39]	Clinical protocols and guidelines	[25]	During the pandemic care duration of treatment pathways was shortened by adjusting protocols to reduce patient traffic.
		Patient volume per day	[12]	Type of care, patient characteristics, e.g., frailty, turnaround time between patients, amount of administration & staff training and education	[12]	Patient volume per day affected the average amount of staff travel per case. This patient volume depended on the type of care and patient characteristics, with less-complex cases and procedures without general anesthetics, leading to higher patient volumes per day. The patient volume per day was also associated with the turnaround time between patients, which could be influenced by staff training and education, but was also described to be dependent on the amount of administration.
		Care frequency	[42]	Type of care	[8,42]	Certain types of care require more or less frequent monitoring and treatment visits.
		Technological developments and improvements	[44]			The development of long-acting agents could reduce the number of injections performed and travel frequency.

### 3.2 Factors related to the carbon footprint of facilities and equipment

**Fig. 2:**
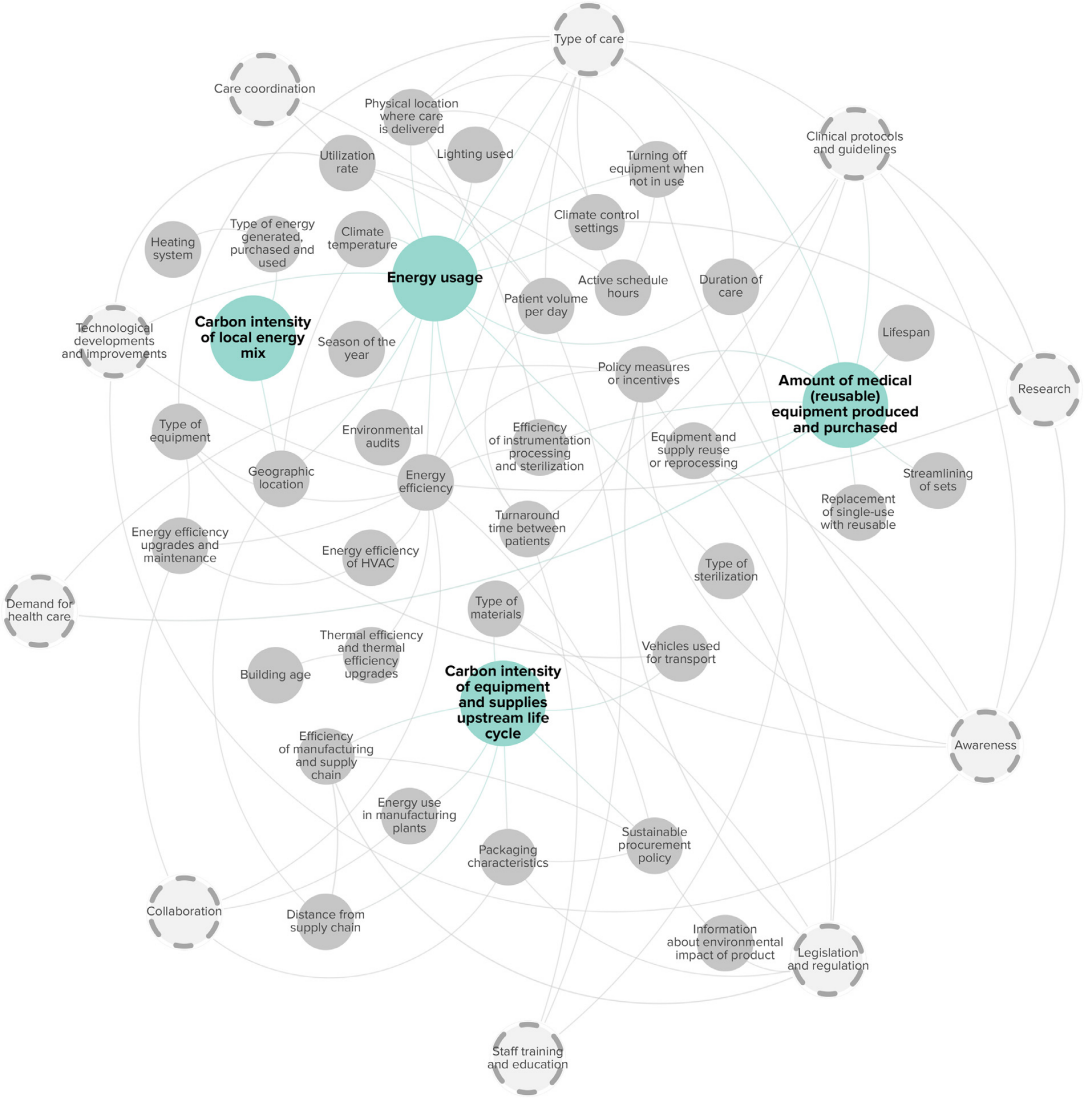
Factors associated with the carbon footprint of facilities and equipment. Main factors are highlighted in blue. Grey circles indicate subfactors, and dashed circles highlight cross-cutting factors.

The carbon footprint related to equipment and facilities was influenced by four main factors: 1) Energy usage; 2) Carbon intensity of the local energy mix; 3) Amount of medical (reusable) equipment produced and purchased; and 4) Carbon intensity of equipment and supplies’ upstream life cycle. Fig. 2 displays their related subfactors and interrelationships.

#### 3.2.1 Energy usage

The energy consumption for hospital services and care pathways is influenced by several subfactors. Energy efficiency plays an important role [21,22,30,33,34,36,[46], [47], [48], [49], [50], [51], [52], [53], [54], [55], [56]]. Energy efficiency is influenced by Heating Ventilation and Cooling (HVAC) systems [22,55], other types of equipment including lighting [19,34,36,37,39,47,49,53,55], building age [21,33,46,51], and efficiency upgrades and maintenance [34,46,49,52,54,55]. Adjusting climate control settings, for instance to match active schedule hours, can reduce energy usage [32,51,53,57], but the feasibility to change HVAC settings also depends on the location within the hospital where care is delivered, the type of care [27,49,51,58], and research evidence for infection prevention and control [53]. Furthermore, turning off imaging equipment, computers, and lighting outside of active schedule hours further reduces energy use [19,32,34,49,50,58], but may not be feasible for all care locations [50,59]. The type [27,32,49,52,58], and duration of care including turnaround time between patients also influence energy usage per service [32,35,36,47,48,60,61], which depend on clinical protocols and guidelines [35], staff training and education [35]. Efficient coordination of care [22,50], and longer active schedule hours [10,49,56], could increase utilization rates and patient volume per day [19,21,22,32,36,50], and reduce energy consumption per service or patient, since energy to operate the equipment and to maintain hospital spaces is divided over a larger number of services. Moreover, sustainable procurement policies [19,55,56], and environmental audits [33,34,62], have the potential to lower energy use. Finally, various external subfactors, including geographic location [33,49,63], climate temperature [10,49], and season of the year [60], also influence energy consumption.

#### 3.2.2 Carbon intensity of local energy mix

The carbon intensity of a hospital's energy mix is influenced by geographic location [21,22,27,35,41,46,55,[63], [64], [65]], and the type of energy generated, purchased and used by hospitals [11,12,28,[32], [33], [34], [35],^37^,^41^,^46^,^49^,^51^,^52^,^54^,^55^,^62^,^66^], which is also dependent on the type of heating system [12,33]. Hospital care in regions that rely more heavily on fossil fuels for electricity production, have a higher carbon intensity [35,63], while countries with more decarbonized electric grids, such as Sweden and France, show lower emissions [21,22,27,55].

#### 3.2.3 Amount of medical (reusable) equipment produced and purchased

Medical reusable equipment encompasses a wide range of items, including surgical packs, gowns, and drapes, as well as larger electronic devices like magnetic resonance imaging (MRI) scanners and hemodialysis machines. The amount of medical equipment is influenced by the type of care [47,60]. For example, simpler procedures often require less instrumentation [47]. Shorter duration of autoclave cycles and improved efficiency of processing instrumentation could lower the number of instruments required to meet daily demand [35], whereas reduced demand for clinical services may lead to decommissioning of capital equipment [67]. In addition, streamlining surgical instrument sets [34], and lengthening their lifespans could lower equipment needs [36,39,53], whereas replacing more single-use items with reusable alternatives would increase the amount of reusable equipment produced and purchased, but could lower the carbon footprint of hospital care [34,53,64,68].

#### 3.2.4 Carbon intensity of equipment and supplies’ upstream life cycle

The type of materials used in equipment and supplies [21,29,37,52,53,68,69], and packaging characteristics, such as the amount and materials used [29,35,37,38,57,65,68], influence the carbon intensity of their upstream life cycle, and can be impacted by policy measures and incentives [52], legislation and regulation [29,38,57,68], and collaboration with manufacturers [35,38,65]. Several studies suggested that legislation and regulation could also play a role by obligating manufacturers to map and publish information about the environmental impact of products [27,29,57,63,68], which could be used as criteria in sustainable procurement policies [8,12,27,38,54,55,57,68]. Furthermore, various supply chain factors, including energy use in manufacturing plants [41], overall efficiency of manufacturing processes [37,38,64,70], the distance from the supply chain [12,21,29,37,41,57,69], and the types of vehicles used for transportation [37,69] influence the carbon intensity of equipment and supplies upstream life cycle. Table 3 provides a detailed description of the factors related to the carbon footprint of facilities and equipment.

**Table 3:** Factors and subfactors linked with facilities and equipment.

Category	Contributing factor	Subfactor	Reference(s)	Associated subfactor(s)	Reference(s)	Description
Facilities and equipment	Energy usage	Climate control settings	[27,32,34,49,[51], [52], [53],^57^,^58^]	Active schedule hours	[32,51,53,57]	By linking climate control settings to active schedule hours, instituting set-back programs, and utilizing low activity modes, health care facilities could reduce energy consumption and associated greenhouse gas emissions.
				Physical location where care is delivered & type of care	[27,49,51,58]	Climate control settings were dependent on the physical location within the hospital where care was delivered, and the type of care provided, with specific requirements for care in operating rooms or intensive care units. Some care could be provided at-home, more unified disinfection or HVAC processes in hospitals could result in lower associated greenhouse gas emissions.
				Research	[53]	Climate control settings in hospitals should be influenced by research evidence for infection prevention and control.
		Climate temperature	[10,49]	Geographic location	[33,49,63]	Differences in the temperature of climates between different geographic locations were related to substantial variations in energy consumption for heating and cooling purposes.
				Season of the year	[60]	The season of the year could affect energy usage in health care facilities. During the summer, hospitals may experience higher energy demands due to increased air conditioning requirements to maintain comfortable temperatures indoors.
		Efficiency of instrumentation processing and sterilization	[35,47]	Type of sterilization, legislation and regulation & physical location where care is delivered	[35,65]	The efficiency of instrumentation processing and sterilization has an impact on energy usage and may differ depending on the physical location of care delivery (e.g., at-home vs. in-hospital). Shorter autoclave cycles reduced per-case energy consumption and improved efficiency of processing instrumentation. The type of sterilization process was influenced by legislation and regulation, since regulatory bodies may restrict certain types of sterilization, such as flash autoclaving, with the purpose of increasing safety while adding energy burdens to the process.
		Energy efficiency	[21,22,30,[32], [33], [34],^36^,[46], [47], [48], [49], [50], [51], [52], [53], [54], [55], [56],^67^]	Energy efficiency of HVAC, type of equipment, energy efficiency upgrades and maintenance, collaboration & research	[19,22,32,34,36,37,39,46,47,49,52,53,55]	Energy efficiency of HVAC or other types of equipment could be improved by energy efficiency upgrades or maintenance, for example through collaboration between medical professionals and the engineering departments in hospital. Research findings about energy usage of equipment could also lead to conversations with manufacturers and the possible incorporation of low-energy standby features in future designs.
				Thermal efficiency and thermal efficiency upgrades & building age	[21,33,34,46,51,52,54]	Newly built operating rooms had more efficient thermal insulation resulting in lower emissions associated with energy usage.
				Awareness, policy measures and incentives & technological developments and improvements	[19,33,36,37,50,52,53,67]	Awareness raising initiatives among health care workers, patients, policymakers, and manufacturers about energy efficiency, could potentially lead to policy measures or incentives that in turn improve energy efficiency. Manufacturers could focus on developing or improving existing technologies, for example energy-efficient design features of ICT and dialysis machines. Other technological developments and improvements included the implementation of occupancy sensors.
		Turning off equipment when not in use	[19,32,34,49,50,58]	Physical location where care is delivered & active schedule hours	[49,50,59]	Efforts to (partially) turn off imaging equipment, computers, and lighting outside of active schedule hours and during times of low demand, such as overnight or weekends, could lead to reductions in energy use. However, certain physical locations did not allow certain medical equipment to be turned off when not in use, for example imaging equipment in hospitals that was required to remain ready for emergency imaging during night hours and weekends, leaving equipment running idle and consuming standby power for extended periods.
				Awareness	[57]	Awareness raising initiatives among health care workers could lead to more frequent turning off lights and equipment when not in use.
		Environmental audits, e.g., waste, energy	[33,34,62]			Environmental audits, including auditing the electricity and water bill to find areas of improvement, and proper energy auditing through submetering of buildings and equipment could guide reduction strategies and practices, leading to more efficient energy use.
		Physical location where care is delivered	[30,51]			Characteristics of the physical location where care is delivered, such as the size of exam rooms or operating theatres are correlated with energy usage.
		Sustainable procurement policy	[19,55,56]			Sustainable procurement policies could lead to the purchasing and use of more energy efficient equipment. For instance, sustainable procurement policies could include the use of standby power usage as a decision criterion and procurement officers could choose to purchase equipment and lighting with less power consumption.
		Type of care	[27,32,49,52,58]	Lighting used	[52]	The type of care, e.g., choice of surgical modality influenced the amount of energy consumed, with simpler procedures typically requiring fewer instrumentation, allowing for more efficient processing of instrumentation, and laparoscopic or robotic procedures requiring minimal lighting.
				Duration of care & clinical protocols and guidelines	[32,35,36,47,48,60,61]	The type of care was also associated with duration of care, for instance the length of stay or the meeting duration for virtual care. More complex types of care required more time, and increased energy usage due to prolonged use of medical equipment and facilities. Strict protocols could shorten surgical duration and decrease energy use per procedure.
		Turnaround time between patients	[35]	Clinical protocols and guidelines & staff training and education	[35]	Staff training and strict clinical protocols and guidelines for sterilization could shorten turnaround time between patients in the operating room and reduce per-case electricity use.
		Utilization rate and patient volume per day	[19,21,22,32,36,50]	Care coordination, active schedule hours & technological developments and improvements	[10,22,32,49,50,56,59]	Efficient coordination of care and longer active schedule hours could increase utilization rates and patient volume per day, and reduce energy consumption of equipment such as MRI and CT scanners for each service, given that energy to operate the equipment and to maintain the space in which care was delivered was divided over a larger number of services. The development of point-of-care analyzers could substitute for larger equipment and improve utilization rates.
	Carbon intensity of local energy mix	Geographic location	[21,22,27,35,41,46,55,[63], [64], [65]]			The geographic location of hospital facilities was related to the carbon intensity of local energy mixes. Studies in regions that relied more heavily on fossil fuels for electricity production, had a higher carbon intensity of their local energy mix. Additionally, decarbonization of electric grids, as seen in countries like Sweden and France with greater shares of low-carbon electricity, resulted in lower emissions associated with energy usage in other studies.
		Type of energy generated, purchased, and used	[11,12,28,[32], [33], [34], [35],^37^,^41^,^46^,^49^,^51^,^52^,^54^,^55^,^62^,^66^]	Heating system	[12,33]	The type of energy generated, purchased, and used by hospitals also influenced the carbon intensity of the local energy mix. By selecting or transitioning to renewable energy sources like hydroelectricity and photovoltaic solar panels, hospitals could reduce greenhouse gas emissions associated with electricity generation. The choice of heating system, such as electric heat pumps versus natural gas or the use of coal boilers, influenced the type of energy purchased and used. For instance, using high-efficiency electric heat pumps instead of natural gas for heating could contribute to lower carbon emissions.
	Amount of medical (reusable) equipment produced and purchased	Demand for health care	[67]			Reduced demand for laboratory services could potentially lead to decommissioning of capital equipment.
		Efficiency of instrumentation processing and sterilization	[35]			Shorter autoclave cycles and improved efficiency of processing instrumentation influenced the number of instruments required to meet daily demand.
		Lifespan	[36,39,53]			The assumed lifespan of equipment could potentially influence the amount of medical equipment produced and purchased.
		Policy measures and incentives	[39]	Legislation and regulation	[39]	Certain policy measures, such as data deletion policy, helps reduce the number of servers needed for data storage, which is also subject to legal requirements that mandate a specific retention period for medical data.
		Replacement of single use with reusable	[34,53,64,68]			Replacement of single-use medical disposables with reusable alternatives could influence the amount of medical equipment purchased and used.
		Streamlining of sets	[34,37]			Streamlining of surgical instrument sets could lower the amount of medical equipment purchased and used.
		Type of care	[47,60]			The specific type of care that was provided influenced the amount of medical equipment necessary. For example, simpler procedures typically required fewer instrumentation, which allowed for more efficient processing of instrumentation, and robot-assisted surgery used more hybrid instruments that are mainly reusable.
	Carbon intensity of equipment and supplies’ upstream life cycle	Distance from supply chain	[12,21,29,37,41,57,69]	Geographic location	[41]	The distance from the supply chain impacted the carbon intensity of equipment and supplies upstream life cycle. However, the possibility to choose for local manufacturers also depended on the geographic location of medical and pharmaceutical supply areas.
		Efficiency of manufacturing and supply chain	[37,38,70]	Distance from supply chain	[37]	Improved efficiency of manufacturing processes in instrument manufacturers’ supply chain could reduce the carbon intensity of equipment and supplies upstream life cycle.
		Energy use in manufacturing plants	[41]	Collaboration	[41]	A substantial portion of products’ greenhouse gas emissions originated from energy use in manufacturing plants, which could potentially be addressed through collaboration and partnerships with producers.
		Information about environmental impact of product	[27,29,57,63,68]	Legislation and regulation	[27,29,63,68]	More information about the environmental impact of products could lead to a lower carbon footprint. Legislation and regulation could play a role by obligating manufacturers to map and publish their associated carbon impacts within their specification documents, or with graphical indicators, alongside any technical specifications.
		Packaging characteristics, e.g., amount and materials	[29,35,37,38,57,65,68]	Legislation and regulation, collaboration	[29,35,38,57,65,68]	Packaging characteristics, such as the amount and materials used, had an impact on the carbon intensity of equipment and supplies upstream life cycle. Packaging characteristics were influenced by legislation and regulation but could potentially be changed by collaborating with manufacturers.
		Sustainable procurement policy	[8,12,27,38,54,55,57,68]			Adopting sustainable procurement policies, in which the environmental impacts of products are considered, could reduce the carbon intensity of equipment and supplies throughout their entire life cycle.
		Type of materials	[21,29,37,52,53,68,69]	Policy measures or incentives, legislation, and regulation & awareness	[29,52]	The type of materials used in medical equipment and supplies impacted the carbon intensity of their upstream life cycle. For instance, catheter components and patches containing certain metals, such as copper, contributed significantly to greenhouse gas emissions, and high-quality polymers used in single-use items also increased their environmental impact. Policy measures and incentives, legislation and regulation, and awareness amongst manufacturers could change the type of materials used.
		Vehicles used for transport	[37,69]	Type of equipment	[37]	The choice of vehicles used for transport was important, with conventional diesel vehicles contributing to higher emissions compared to electric vehicles, and air transportation by cargo planes leading to more emissions than sea freighters. The choice of vehicle may also depend on the type of equipment since the supply of medical equipment may sometimes require urgent transfers of products by air.

### 3.3 Factors related to the carbon footprint of medical disposables and waste disposal

**Fig. 3:**
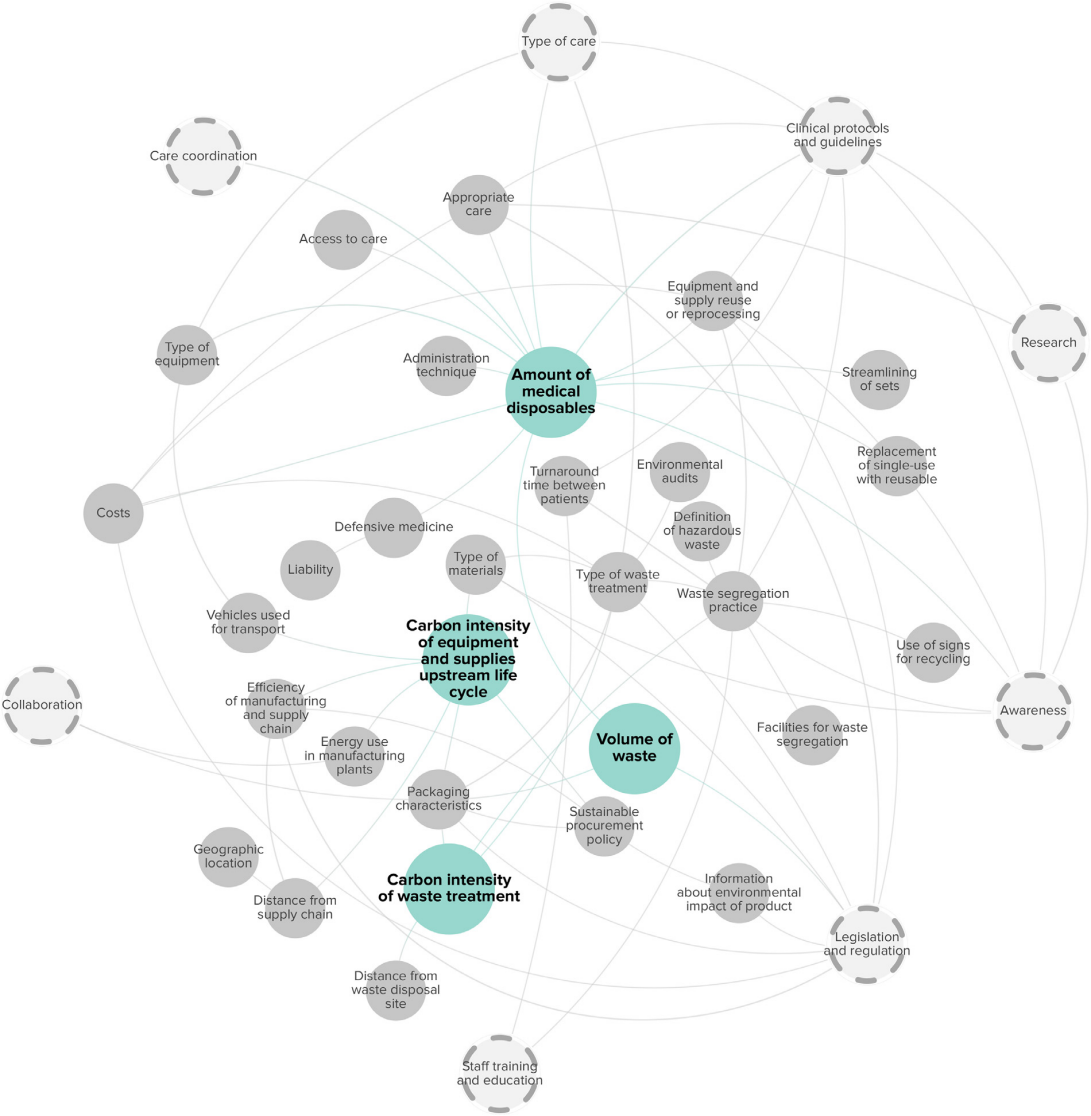
Factors associated with the carbon footprint of medical disposables and waste disposal. Main factors are highlighted in blue. Grey circles indicate subfactors, and dashed circles highlight cross-cutting factors.

The carbon footprint related to medical disposables was influenced by two main factors: 1) Carbon intensity of equipment and supplies’ upstream life cycle; 2) Amount of medical disposables. The carbon footprint related to waste disposal was influenced by two main factors: 1) Volume of waste; 2) Carbon intensity of waste treatment. The Carbon intensity of equipment and supplies’ upstream life cycle was described in the previous section, as the same subfactors applied to reusable and single-use medical items’ upstream life cycle. The factors associated with the amount of medical disposables and waste disposal are described in the next sections and are displayed in Fig. 3.

#### 3.3.1 Amount of medical disposables

The type of care often determines the need for a certain amount of medical disposables, for example advanced procedures typically requiring more single-use accessories [27]. However, appropriate care practices, such as the selective use of infusion in case of clinical signs of hypovolemia [27,68], and raising awareness can lead to disposable savings [57]. Replacing single-use medical disposables with reusable alternatives [34,53,64,68], and equipment and supply reuse or reprocessing [8,11,12,35,52,54,55,58,68], can reduce the amount of medical disposables produced and purchased, but depends on clinical protocols and guidelines, research evidence for infection prevention and control [8,21,34,35], and legislation and regulation [54]. Recommendations regarding sterility practices [21,34,55], and defensive medicine [35], often lead to increased, and in some cases unnecessary, single-use supply use. Streamlining of disposable sets [11,29,44,53,55,58,64], standardizing administration techniques [29,31], and coordination of care can also lower the amount of disposables [71].

#### 3.3.2 Volume of waste

The volume of waste is closely linked to the number of medical disposables. Subfactors that influence the production and use of single-use items directly impact waste levels. In addition, effective waste segregation practices, which differentiate between contaminated and non-contaminated supplies, can reduce waste by allowing more items to be reused [35]. Additionally, clinical protocols and guidelines and optimized stocking systems can prevent unnecessary waste [55].

#### 3.3.3 Carbon intensity of waste treatment

The carbon intensity of waste treatment is influenced by the type of waste treatment [11,12,21,22,26,[33], [34], [35],^37^,^41^,^44^,^49^,^55^,^57^,^58^,^64^,^69^,^71^,^72^], such as recycling or incineration, and waste segregation practices [29,37,38,51,55,57,58,64,71,73], which depend on the presence of facilities and waste containers for recycling [55], the use of signs for recycling and educating staff [55,71], the implementation of strict hazardous waste protocols [37,73], the definition of what is considered to be hazardous [51], and legislation and regulation [21,57]. Table 4 provides a detailed description of the factors related to the carbon footprint of medical disposables and waste disposal.

**Table 4:** Factors and subfactors linked with medical disposables and waste disposal.

Category	Contributing factor	Subfactor	Reference(s)	Associated subfactor(s)	Reference(s)	Description
Medical disposables and waste disposal	Carbon intensity of equipment and supplies’ upstream life cycle	*See*Table 2.				
	Amount of medical disposables	Access to care	[8]			Single-use, pre-packaged products have facilitated increased access to dialysis treatment over time.
		Administration technique	[31]			The amount of medical disposables produced and purchased could also be reduced by standardizing administration techniques.
		Appropriate care	[27,68]			Appropriate care practices, such as the selective use of infusion in case of clinical signs of hypovolemia, had the potential to lower carbon emissions, including those from the production and disposal of medical supplies.
		Awareness	[57]			Raising awareness among healthcare workers, patients, policymakers, and manufacturers about sustainable practices, could lead to disposable savings.
		Care coordination	[71]			Appropriate care planning could prevent excess and the inadvertent use of disposable accessories.
		Clinical protocols and guidelines	[29]			Consumable use could be rationalized by using pre-set lists.
		Defensive medicine	[35]	Liability	[35]	Excessive resource use was also driven by defensive medicine, because of liability concerns.
		Equipment and supply reuse or reprocessing	[8,11,12,35,52,54,55,58,68]	Clinical protocols and guidelines, research, legislation and regulation, costs.	[8,21,34,35,54]	The reuse of equipment and supplies could lower demand for disposable items but is influenced by clinical protocols and guidelines and research evidence for infection prevention and control, legislation and regulation. Recommendations regarding sterility practices often led to increased, and in some cases unnecessary, single-use supply use. Reuse of equipment could also lead to decreased costs.
		Replacement of single use with reusable	[34,53,64,68]			The replacement of single-use medical disposables with reusable alternatives lowers the amount of consumables used.
		Streamlining of sets	[11,29,44,53,55,58,64]			Streamlining of disposable sets could have an impact on greenhouse gas emissions associated with their production and usage. By redesigning procedure packs to minimize unnecessary single-use items and optimizing them to include only essential or lighter-weighted materials, the amount of medical disposables purchased and produced could be reduced.
		Type of care	[27]			Certain advanced procedures required a high number of single-use accessories.
		Type of equipment	[59]			The type of equipment also influences the amount of consumables, for example certain analyzers require a minimum volume of blood for testing, driving the size of collection tubes.
	Volume of waste	*See*Table 3*for subfactors described under ‘Amount of medical disposables’.*				
		Clinical protocols and guidelines	[55]			Clinical protocols and guidelines specific to stocking of supplies in patient rooms have the potential to avoid unnecessary waste of supplies, since supplies in some patient rooms have to be disposed after every discharge or transfer.
		Packaging characteristics, e.g., amount and materials	[55]			The amounts and size of packaging influences the volume of waste generated.
		Waste segregation practice	[35]			Good waste segregation practices and delineating between contaminated and non-contaminated surgical supplies, had an impact on the amount of supplies that could be reused after proper sorting lowering waste volumes.
	Carbon intensity of waste treatment	Distance from waste disposal site	[11,12,33]			The distance from waste disposal sites affects the carbon intensity of waste treatment. Disposing of waste locally reduces emissions associated with transportation.
		Type of waste treatment	[11,12,21,22,26,[33], [34], [35],^37^,^41^,^44^,^49^,^55^,^57^,^58^,^64^,^69^,^71^,^72^]	Legislation and regulation, waste segregation practice, facilities for waste segregation, turnaround time between patients, clinical protocols and guidelines, definition of hazardous waste, awareness, staff training and education & use of signs for recycling	[21,29,33,37,38,51,55,57,58,64,71,73,91]	Different types of waste treatment, such as recycling or incineration, each carry their own carbon intensity. The feasibility of different types of waste treatment was influenced by legislation and regulation, and waste segregation practices, such as delineating between contaminated and non-contaminated waste. Proper sorting can only be done if the right facilities for waste segregation are available, such as waste containers for recyclables, and if time between procedures allows for proper waste sorting. For certain types of waste, such as hazardous waste, waste segregation practices also depended on the definition of what was considered to be hazardous, and the implementation of strict hazardous waste protocols. A lack of awareness on appropriate waste segregation practices can be a barrier, but staff training and education, and the use of signs for recycling can improve these practices and reduce certain types of waste.
				Type of care, type of materials, packaging characteristics, sustainable procurement policy & legislation and regulation	[33,57]	The type of care, materials and packaging characteristics impact the type of waste treatment. For example, the type of care influences the materials needed, and many materials have a low-recycling fraction. Sustainable procurement policy could prioritize ordering items with recyclable packaging, and legislation and regulation could impose norms for higher recycling fractions.
				Environmental audits, e.g., waste, energy	[33]	Environmental audits, including waste audits can help find areas of improvement and lower carbon intensity of waste treatment.
				Awareness	[38,91]	Awareness amongst staff influences the feasibility of certain type of waste treatment including recycling. Certain types of waste treatment like recycling can in turn also raise awareness on sustainability within healthcare.

### 3.4 Factors related to the carbon footprint of pharmaceuticals

**Fig. 4:**
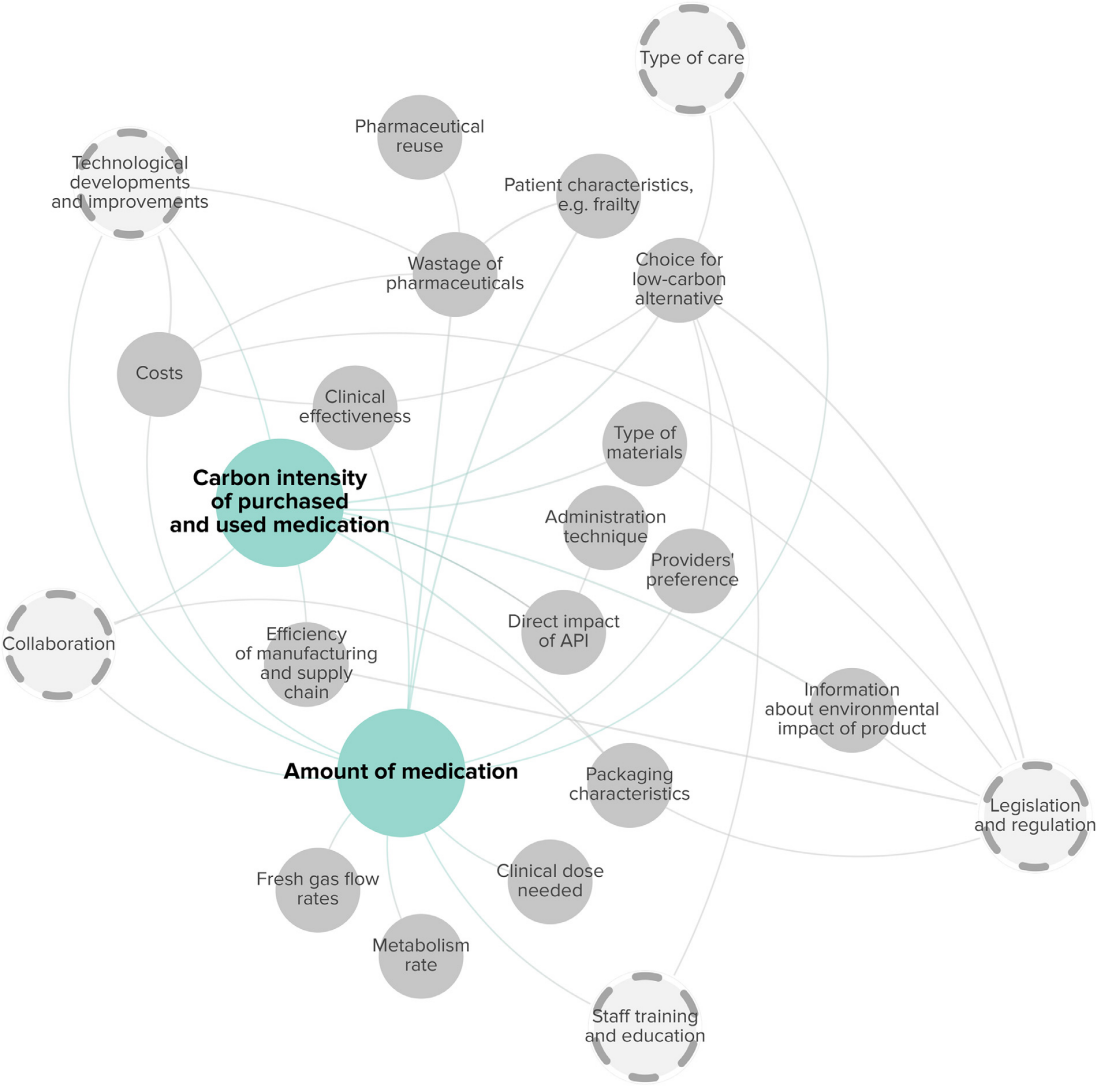
Factors associated with the carbon footprint of pharmaceuticals. Main factors are highlighted in blue. Grey circles indicate subfactors, and dashed circles highlight cross-cutting factors.

The carbon footprint related to pharmaceuticals was influenced by two main factors: 1) Amount of medication; 2) Carbon intensity of purchased and used medication. Fig. 4 displays their related subfactors and interrelationships.

#### 3.4.1 Amount of medication

The amount of medication is influenced by various subfactors, including the clinical dose needed [66], the effectiveness of treatments [70], the type of care [58], flow rates [48,62,66], the rate of metabolism [66], and patient characteristics such as polypharmacy and patient compliance [8]. Wastage of pharmaceuticals also influences the total amount of medication [8,21,35,57,58,62,64,66], but could be improved by efforts to reuse pharmaceuticals (e.g., multiuse pharmaceuticals) [35,58,64], and technological developments and improvements. For instance, advancements such as the installation of waste gas capturing technology [52,66], improving mechanical ventilator efficiency [57], and closed-circuit delivery of anesthesia in operating rooms [62], may mitigate wastage of pharmaceuticals. Next to this, the development and introduction of long-acting pharmaceutical agents may reduce both emissions and costs associated with medication use [44].

#### 3.4.2 Carbon intensity of purchased and used medication

Some active pharmaceutical ingredients (APIs) have a high carbon impact, such as desflurane and nitrous oxide (N_2_O) [52,58,66]. Opting for low-carbon alternatives, when clinically appropriate, can reduce emissions [29,51,52,62], though costs [62] and providers' preference [51,52] also play a role. Improved efficiency of manufacturing processes in pharmaceutical manufacturers’ supply chain can lower the carbon intensity of medication's upstream life cycle [70]. Factors previously mentioned to be important for the carbon intensity of equipment and supplies’ upstream life cycle, including packaging characteristics and information about the environmental impact of products, were also found to be important for pharmaceuticals [29]. Table 5 provides a detailed description of the factors related to the carbon footprint of pharmaceuticals.

**Table 5:** Factors and subfactors linked with pharmaceuticals.

Category	Contributing factor	Subfactor	Reference(s)	Associated subfactor(s)	Reference(s)	Description
Pharmaceuticals	Amount of medication	Clinical dose needed	[66]			The clinical dose that was needed for effective treatment impacted the amount of medication used. For instance, drugs with lower anesthetic potency require larger doses, leading to increased medication usage and environmental impact.
		Clinical effectiveness	[70]			Clinical effectiveness also played a role in determining the amount of medication used. For example, a better success rate of a surgical procedure to resolve complaints could lower the need for medication use.
		Collaboration	[92]			Good collaboration between surgical and anesthesia staff could influence the amount of medication given during surgery, especially towards the patient emergence at the end of a surgical procedure.
		Fresh gas flow rates	[48,62,66]			For anesthesia specifically, three studies described how fresh gas flow rates during anesthesia impact the amount of medication used.
		Metabolism rate	[66]			The rate of metabolism affected the amount of medication required for anesthesia administration. For instance, anesthetic agents with low rates of metabolism, like desflurane, may result in larger proportions of the gas escaping into the atmosphere, contributing to higher greenhouse gas emissions.
		Patient characteristics, e.g., frailty	[8]			Patient characteristics, such as polypharmacy and patient compliance have an influence on the amount of medication used.
		Providers' preference	[92]			Provider preferences seemed to influence the amount of medication given during surgery.
		Technological developments and improvements	[44]	Costs	[44]	The development and introduction of long-acting pharmaceutical agents may reduce the amount of medication use.
		Type of care	[58]			The type of care was related to the amount of medication used. For example, certain types of care, such as interventions may be performed with or without general anesthetics, influencing the amount of medication given.
		Wastage of pharmaceuticals	[8,21,35,57,58,62,64,66]	Pharmaceutical reuse, costs & policy measures, or incentives	[35,58,64]	Wastage of pharmaceuticals also impacted the total amount of medication and could be influenced by efforts to reuse pharmaceuticals where feasible (e.g., multiuse pharmaceuticals), which would also lower associated costs.
				Technological developments and improvements	[52,57,62,66]	Technological developments and improvements have the potential to impact wastage of pharmaceuticals. For instance, advancements such as the installation of waste gas capturing technology, improving mechanical ventilator efficiency, and closed-circuit delivery of anesthesia in operating rooms, may mitigate wastage of pharmaceuticals.
	Carbon intensity of purchased and used medication	Administration technique	[66]			The administration technique affected the carbon intensity of purchased and used medication. For example, the choice for using N2O/O2 as carrier gas during procedures influenced the carbon intensity of medication usage.
		Choice for low-carbon alternative	[29,51,52,62]	Type of care, costs, providers' preference & staff training and education	[51,52,62]	When clinically appropriate, choices for low-carbon alternatives, including anesthetics, could reduce carbon footprint of hospital care delivery. Some of these choices also influence the type of care provided, for example when a different imaging modality is used. Choices for low-carbon alternatives are also related to costs, and providers' preference, which could be influenced by staff training and education.
		Collaboration	[38]			Collaboration between the pharmaceutical industry and healthcare professionals could reduce emissions in the value chain.
		Direct impact of API	[52,58,66]			The direct impact of APIs, such as desflurane and nitrous oxide (N2O), significantly influenced the carbon intensity of purchased and used medication
		Efficiency of manufacturing and supply chain	[70]			Improved efficiency of manufacturing processes in pharmaceutical manufacturers’ supply chain could reduce the carbon intensity of medication's upstream life cycle.
		Information about environmental impact of product	[29]	Legislation and regulation	[29]	Information about the environmental impact of pharmaceutical products could lead to a lower carbon footprint. Legislation and regulation could encourage manufacturers to label their products.
		Packaging characteristics, e.g., amount and materials	[29]	Legislation and regulation	[29]	A reduction of packaging and paper instructions for pharmaceuticals, and the development of recycled and or reusable materials could be encouraged by legislation and regulation.

### 3.5 Cross-cutting factors

Amongst all subfactors, ten factors interacted with multiple elements of the carbon footprint (highlighted in dashed circles, Fig. 1, Fig. 2, Fig. 3, Fig. 4). For example, awareness was associated with the amount of energy and medical disposables used, the carbon intensity of waste treatment, and the mode of transportation chosen by patients and staff. Coordinating care efficiently, for example by planning different appointments on the same day, prioritizing care delivery to the most local clinics, and increasing daily patient volumes and utilization rates, could also lower energy use, travel distance, and travel frequency per service. Collaboration was connected with both the carbon intensity of equipment and supplies' upstream life cycle, the mode of transportation, and energy use of HVAC systems. Collaborating with local governments to subsidize public transport could decrease the carbon intensity of transportation. Internally, fostering collaboration between physicians and the engineering department could enhance the energy efficiency of HVAC systems. The ten factors can be seen as cross-cutting factors, as directed efforts towards these factors have the potential to impact the carbon footprint of hospital care in multiple ways.

## 4 Discussion

This study identified and mapped twelve main factors influencing four major domains of the carbon footprint of hospital services and care pathways: travel, facilities and equipment, consumables and waste disposal, and pharmaceuticals. Each main factor interacted with multiple subfactors, which varied in the level on which they operate, actors involved, complexity, and changeability. It was found that sustainable hospital care practices are influenced by factors operating at different levels. At the micro-level, health care professionals can directly modify the carbon footprint, such as by choosing low-carbon anesthetic options. Meso-level changes, such as green transportation facilities, rely on hospital management's willingness to invest. Macro-level factors, such as national or international legislation and regulation, typically lie beyond direct hospital influence. This complexity emphasizes that a single solution to significantly lower the carbon footprint of hospital care does not exist.

Our study results corroborate factors identified in previous research. A systematic literature review on climate adaptive hospitals found that factors such as energy efficiency, use of renewable energy, the location of the hospital and the promotion of active and alternative travel were important components of climate mitigation measures at the organizational level [74]. Another review reported that renewable energy use and resource usage were important factors that contribute to improving performance of environmental sustainability in health care organizations [75]. Another study on environmental strategies in Italian health care organizations also found that organization-specific barriers for their adoption could be influenced by awareness campaigns, education and the provision of incentives in various forms [76]. Such barriers, including education and awareness, incentives, facilities and equipment, were also identified by a review on barriers for the implementation of sustainable measures specifically in operating theatres [77]. Finally, an adapted realist evaluation study in the UK explained how the type of implemented measures across hospitals was influenced by collaboration with partner organizations and the geographic location of hospital sites [78]. The overlap in factors from these studies and the ones identified in our study suggests that determinants relevant at the organizational level also shape the environmental footprint of individual hospital services. This poses a challenge for the transferability of research results from one setting to another. Others have argued before that variability in clinical practice, procurement and energy sources complicates interpretation across health systems and regions [79]. Our study provides more details into the variety of factors at play of which end-users of results should be cognizant when interpreting carbon footprint results for both individual hospital services and entire care pathways, for which the influence of certain organizational factors could even be greater [80].

### 4.1 Strengths and limitations

To our knowledge, this is the first study to summarize both direct and indirect factors influencing the carbon footprint of individual hospital services and care pathways, grounded in theory, and supported by data from scientific reports that quantified these footprints. With the rapid increase of carbon footprint studies in hospital care, this study seized the opportunity to draw broader insights from these studies to create a more comprehensive understanding of where and how reductions in hospital care's carbon footprint could be achieved. By taking this approach, this study revealed novel important factors that influence hospital services’ carbon footprint that have not been identified by previous studies on sustainability in hospital care [[74], [75], [76],^78^,^81^]. Next to identifying novel factors, our study also mapped interrelationships between factors, and the created visual maps show how certain factors are highly interrelated with different parts of hospital care's carbon footprint. Among these, ten cross-cutting factors have been identified as important system-level target areas that can be leveraged to accelerate the transition to carbon-neutral hospital care. Finally, our study contributes to a better understanding of causes of variation of hospital services and care pathways, which is important for improving transferability of results and conclusions in this exponentially growing research domain [82].

Our study specifically examined factors related to the climate impact of hospital care, distinct from broader environmental sustainability issues [75,76,81,83]. This allowed for an in-depth exploration of contributing factors and their interrelations within a manageable scope. However, the health care sector also affects other environmental outcomes, such as resource consumption, water pollution, and land use [3]. Future research should address these additional environmental outcomes to develop more comprehensive sustainability strategies. Our analysis was based primarily on the discussion sections of articles, which reflect authors’ interpretations on the factors affecting the carbon footprint of the hospital services and care pathways they studied. This approach was necessary due to the lack of empirical studies on these determinants. While qualitative analysis can reveal valuable insights about plausible causes and probable outcomes [84], it may not capture all relevant interrelationships, and the causal impact of modifying certain factors remains uncertain. Additionally, our exploratory study did not provide quantitative information on strength and direction of relationships between factors. To gain a deeper understanding, future research should include expert interviews and other primary data sources.

### 4.2 Conclusion

This study provides insight into the carbon footprint of hospital services and care pathways by identifying and mapping over eighty relevant factors, that help guide targeted actions for reducing carbon emissions of hospital care. The identified factors were derived from environmental footprint research designed to help stakeholders find relevant carbon reduction areas for specific hospital services, care pathways and settings, and track progress. Given the variability in carbon footprints across different settings, these localized studies are important to adapt sustainability efforts to their local contexts. Furthermore, evidenced-based information on the influence of specific factors, such as the replacement of single-use with reusable, challenges current practices and beliefs, and is crucial in the transition to more sustainable healthcare [85]. Our study also recognizes research as one of ten cross-cutting factors influencing the environmental impact of health care in various ways. However, while environmental sustainability measurements are valuable, detailed assessments are resource- and time-intensive [86], and face numerous challenges, including the lack of harmonized definitions and calculation methods [9,87], misalignment of metrics [86], and varying quality [9,14]. Furthermore, within this study theoretical saturation was reached, with many carbon footprint studies suggesting similar factors and measures. Therefore, research efforts should not only focus on measuring environmental impact with the aim to identify potential mitigation measures, but also on implementing interventions that address the most significant factors. Our study suggests ten cross-cutting factors that can be leveraged by stakeholders to lower the carbon footprint of hospital care in multiple ways, but future research should evaluate whether these are indeed most impactful, while accounting for the complexity and non-linear relationships between variables. Complexity science, also called systems thinking, highlights the need to view hospital care as a complex system where interactions and feedback loops influence outcomes [[88], [89], [90]]. Future research should prioritize understanding these interactions and behavior patterns over time, rather than studying the impact of altering any factor in isolation.

## Funding

This project was supported by a grant from APH Research Institute. The funder of the study had no role in study design, data collection, data analysis, data interpretation, or writing of the report.

## CRediT authorship contribution statement

**L.H.J.A. Kouwenberg:** Writing – review & editing, Writing – original draft, Project administration, Methodology, Investigation, Formal analysis, Data curation, Conceptualization. **D.S. Kringos:** Writing – review & editing, Supervision, Methodology, Data curation, Conceptualization, Funding acquisition. **W.J.K. Hehenkamp:** Writing – review & editing, Data curation, Conceptualization. **E.S. Cohen:** Writing – review & editing, Investigation, Data curation. **N.H. Sperna Weiland:** Writing – review & editing, Supervision, Methodology, Funding acquisition, Data curation, Conceptualization.

## Declaration of competing interest

The authors declare that they have no known competing financial interests or personal relationships that could have appeared to influence the work reported in this paper.

## Appendix Supplementary materials

Image, application 1

Image, application 2
