# Supplementary material for: The interplay of factors influencing the carbon footprint of hospital care—A causal mapping analysis of scientific reports
Source: J Clim Chang Health. 2025 Mar 12;23:100427. doi: 10.1016/j.joclim.2025.100427 (PMC12851210; doi:10.1016/j.joclim.2025.100427)
Supplement: Supplementary file 1 [file mmc1.docx]

Submission Requirements The Journal of Climate Change and Health:

Note to authors: This form must be completed in full and uploaded with your submission: incomplete answers will result in delay in review of your submission and return to the author for correction

✓ I have read the Guide for Authors and followed its recommendations

✓ The cover letter is complete and includes

✓ The type of article

✓ Whether it is intended for a Special Issue

✓ The word count for text (not including references, figures, tables or abstract)

✓ The abstract is limited to 250 words

✓ The abstract abides by the following standards (check one)

✓ Research Articles and Reviews require a structured abstract with Introduction, Methods, Results, and Conclusions

- - Short Communications, Case Reports, and Perspectives require an unstructured abstract.

✓ The article, according to the standards described in the Guide for Authors, follows the standards based on article type for word count, reference number and format including numbering of sections

✓ In-text citations are bracketed and in Arabic numerals [ ]

✓ In-text citations come before punctuation at the end of a sentence

✓ Language/grammar has been checked by a primary English speaker if you are not a primary English-speaker

✓ Language/grammar is in American English

✓ A potential reviewer is listed with their email address

✓ References are complete/accurate, in Vancouver format and in accordance with the

Journal of Climate Change and Health’s information to authors

✓ All references in the reference list are cited in the text?

✓ The conflict of interest form titled “The Declaration Tool” completely filled out?
